# The mysterious dialogue between the embryo, endosperm, and seed coat, and its implications for seed traits

**DOI:** 10.1093/jxb/eraf320

**Published:** 2025-07-12

**Authors:** Duarte D Figueiredo, Rita A Sharma

**Affiliations:** Department of Plant Reproductive Biology and Epigenetics, Max Planck Institute of Molecular Plant Physiology, Am Mühlenberg 1, Potsdam 14476, Germany; BRIC-National Agri-Food and Biomanufacturing Institute (NABI), Mohali, Punjab, India; Sorbonne Université, France

**Keywords:** Communication, embryo, endosperm, seed, seed coat, seed quality, seed size

## Abstract

The seeds of flowering plants contain three genetically distinct structures: the embryo, which will form a new plant; the endosperm, which nourishes the embryo; and the seed coat, which protects the embryo and endosperm. For a seed to form, these three structures have to communicate and coordinate their development. This communication is not just necessary for seed viability; it also underlies important agronomic traits like seed size. In this review, we outline the current body of knowledge on how the embryo, endosperm, and seed coat communicate with one another during the early stages of seed development. We also discuss the nature and variability of signalling mechanisms across these tissues and the impact of these interactions on seed development and associated agronomic traits, highlighting how understanding these communication pathways can contribute to agricultural biotechnology.

## Teaser

Understanding the interactions between embryo, endosperm, and seed coat is essential for both advancing our fundamental understanding of seed development and improving agronomic seed traits.

## Introduction

**Fig. 1.**
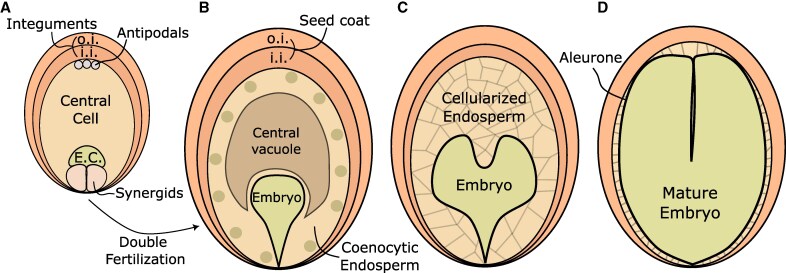
Stages of development for a seed with a nuclear-type transient endosperm. (A) A typical *Polygonum*-like mature ovule contains two gametes, the central cell and egg cell (E.C.), which are accompanied by two synergids and three antipodal cells. These gametophytic cells are surrounded by two integuments, inner (i.i.) and outer (o.i.). (B) Following double fertilization, two products start developing, the embryo and the endosperm. The latter initially develops as a coenocyte, where karyokinesis is uncoupled from cytokinesis, and a large vacuole occupies the center. The seed coat develops from the integuments and protects the fertilization products. (C) Later in development, the coenocytic endosperm cellularizes, after which it starts being consumed by the embryo. (D) In species with transient endosperms, like Arabidopsis, the embryo consumes almost the whole of the endosperm, leaving a single cell layer called aleurone.

The life cycle of plants comprises an alternation between two generations: the diploid sporophyte and the haploid gametophyte. The transitions between these two generations are brought about by meiosis, which reduces the chromosome numbers by half, and by fertilization, which restores the diploid condition. While in bryophytes the gametophytic generation is the dominant one, it became progressively reduced in vascular plants, to the extent that in seed-bearing plants (the spermatophytes), it comprises only a few cells. For instance, in the angiosperms, the male gametophyte, or pollen grain, is composed of three cells: two gametes (or sperm cells) and one vegetative cell. Its maternal counterpart, called the embryo sac, is usually composed of seven cells: two gametes (egg cell and central cell), and five accessory cells (synergids and antipodals) (Fig. 1A). There are variations in the number of female gametophytic cells, but the seven-celled gametophyte, also called the *Polygonum*-type, is the prevalent type in angiosperms, occurring in over 70% of known species (Reiser and Fischer, 1993; Schmid *et al*., 2015). The embryo sacs develop inside the ovule primordia, deeply embedded in the maternal sporophytic tissues, and at maturity are surrounded by the ovule integuments (Schneitz *et al*., 1995; Vijayan *et al*., 2021).

The fertilization of the two maternal gametes by the paternal sperm cells, termed double fertilization, is the event that triggers seed formation and the start of a new sporophytic generation (Dresselhaus *et al*., 2016). After delivery of the sperm cells by the pollen tube, karyogamy takes place between the gametes, and two fertilization products are formed: the embryo, which will form a new plant; and the endosperm, which functions as a nourishing structure (Fig. 1B). These two structures are surrounded by a maternal tissue called the seed coat, which is derived from the ovule integuments (Fig. 1B). In most species, the embryo is diploid and the endosperm triploid, as it results from the fertilization of the homodiploid central cell by a sperm cell. This means that a developing seed contains three genetically distinct structures: the embryo contains one maternal and one paternal genome copies (1M:1P), the endosperm contains two maternal and one paternal genome copies (2M:1P), and the seed coat remains unfertilized, and thus is made up of two maternal genome copies, with no paternal contribution (2M:0P), like the other maternal sporophytic tissues. Importantly, for a viable seed to develop, communication and coordination between these three different structures is essential. In this review, we will cover the current state-of-the-art on how the three seed structures communicate with one another. We first mostly focus on what is known in the model species *Arabidopsis thaliana* (Arabidopsis), and then explore some differences and commonalities in communication between seeds of different clades of angiosperms. We also highlight currently unanswered questions in the field, as well as potential avenues for agricultural applications. To keep the article concise, we mostly focus on early stages of seed development. Stages such as seed maturation and dormancy are not covered in this review.

## The endosperm is indispensable for embryogenesis and its development is shaped by the embryo

In most angiosperms the endosperm initially develops as a coenocyte, that is, in early stages of endosperm formation, there is no *de novo* deposition of cell walls after each mitotic event, meaning that the endosperm is a multinucleate cell (Fig. 1B) (Brown *et al*., 1999; Olsen, 2004). Because the endosperm is mostly occupied by a large central vacuole at this point (Olsen, 2001), it functions as a strong nutrient sink. Then, at a given point in development, the endosperm cellularizes (Fig. 1C), that is, cell walls are deposited surrounding the endosperm nuclei, and the central vacuole is mostly abolished (Olsen, 2001). The exact timing of endosperm cellularization varies from species to species. For example, in Arabidopsis it happens around 5 d after pollination (Batista *et al*., 2019a), when the embryo is at the heart stage, while in rice the endosperm is already at an advanced stage of cellularization at 3 d after pollination (Tonosaki *et al*., 2021). At later stages of development, the embryo grows and consumes part or almost the totality of the endosperm (Fig. 1D).

The endosperm functions as a nourishing structure, and it accumulates nutrients that are later transferred to the embryo or to the germinating seedling. As such, a failure of the endosperm in acquiring nutrients from the mother or in transmitting them to the next generation naturally leads to embryo abortion. Here, we will not focus on the nutritional contribution of the endosperm to the embryo, but rather on other signals that originate in the endosperm and that are required for proper embryogenesis. For example, the peptide CLAVATA3/EMBRYO SURROUNDING REGION-RELATED8 (CLV3/CLE8), which is expressed in early stages of seed development in Arabidopsis, is necessary for non-cell autonomous communication between endosperm and embryo, ensuring proper embryo patterning and endosperm proliferation (Fiume and Fletcher, 2012). Notably, however, the embryo can survive without an accompanying endosperm in its very early stages of development. In Arabidopsis, pollination by fathers mutant for *Cyclin Dependent Kinase A;1* (*CDKA;1*) or for *F-BOX LIKE17* (*FBL17*) often results in single fertilization of the female gametophyte, where only the egg cell is fertilized (Iwakawa *et al*., 2006; Gusti *et al*., 2009). The resulting embryo, although not supported by an endosperm, can still develop until the early globular stage (Iwakawa *et al*., 2006; Gusti *et al*., 2009). Importantly, live imaging indicates that embryo patterning itself is not seemingly affected in those embryos devoid of endosperm (Gooh *et al*., 2015). Rather, it seems that patterning factors for early embryogenesis are provided maternally, and not zygotically. This is because embryogenesis factors present in the endosperm are already present in the central cell of the ovule. Cysteine-rich peptides of the EMBRYO SURROUNDING FACTOR1 (ESF1)-type are required for proper patterning of the Arabidopsis embryo (Costa *et al*., 2014). The knock-down of *ESF1s* leads to defects in the suspensor and the embryo proper, which coincides with the misexpression of several marker genes including *WUSCHEL RELATED HOMEOBOX5* (*WOX5*), *WOX8*, *AUXIN RESPONSE FACTOR 13* (*ARF13*), INDOLEACETIC ACID-INDUCED PROTEIN 10 (*IAA10*), and *PIN-FORMED 1* (*PIN1*), which are critical for apical–basal axis formation, suspensor development and proper auxin distribution (Costa *et al*., 2014). Crossing of *esf1* RNAi female plants with *cdka;1* pollen resulted in single-fertilized globular embryos with severe patterning defects and short suspensors indicating that maternally contributed central ESF1 peptides are necessary for proper embryogenesis (Costa *et al*., 2014).

Although the endosperm seems to be somewhat dispensable for early embryo formation, a viable endosperm is strictly required for embryo survival at later stages of development. For instance, the endosperm of maize *rough endosperm3* (*reh3*) mutants fails to differentiate, which leads to embryo abortion (Fouquet *et al*., 2011). Moreover, if the endosperm fails to cellularize, or the timing of cellularization is delayed, the accompanying embryo dies (Hehenberger *et al*., 2012). This is not just the case in Arabidopsis, but also in other species, like rice (Cheng *et al*., 2021; Tonosaki *et al*., 2021). However, the reason why cellularization of the endosperm is necessary for embryo survival was not immediately clear. Because the coenocytic endosperm functions as a sink for nutrients, it was hypothesized that failure of cellularization would prevent the embryo from becoming the sink tissue, leading to its starvation (reviewed in Lafon-Placette and Köhler, 2014). However, recent findings in Arabidopsis suggest that the cellularized endosperm is required for the establishment of dehydration tolerance in the embryo (Xu *et al*., 2023). Consistent with this, embryos surrounded by non-cellular endosperm have impaired abscisic acid (ABA) responses (Xu *et al*., 2023).

**Fig. 2.**
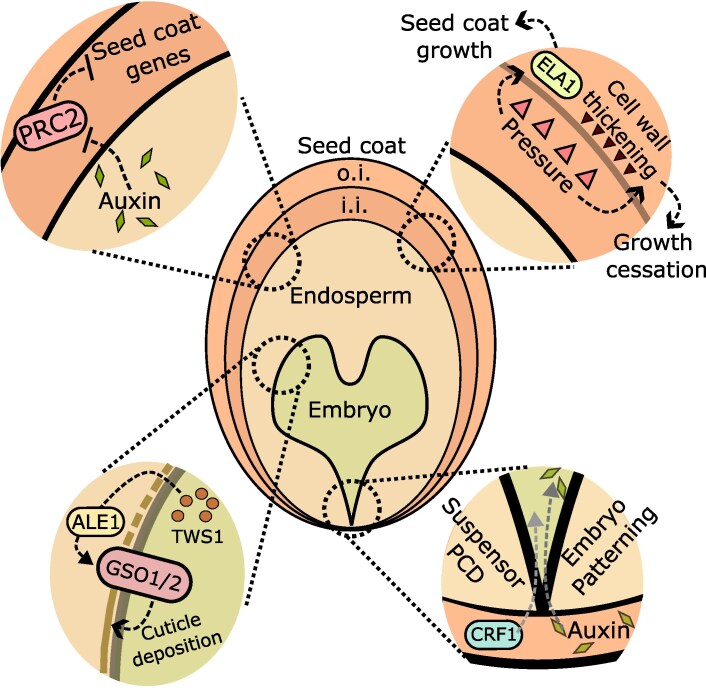
Examples of communication between the three seed structures. Auxin produced in the endosperm is transported to the integuments to remove repressive PRC2s, which repress the expression of genes necessary for seed coat formation in Arabidopsis (upper left panel). Pressure from the zygotic products, embryo and endosperm, is sensed by a mechanosensitive layer of the Arabidopsis seed coat. This initially leads to the up-regulation of ELA1, which promotes seed coat growth. Later in development, this internal zygotic pressure promotes cell thickening of a specific seed coat layer, which contributes to the cessation of seed coat growth (upper right panel). The TWS1 peptide is produced in the embryo, exported to the endosperm where it is cleaved by ALE1. The cleaved product moves back to the embryo where it is perceived by GSO1/2, which mediate *de novo* cuticle deposition in Arabidopsis. When a continuous cuticle is established, TWS1 can no longer travel to the endosperm, and this communication ceases (bottom left panel). Sporophytic signals are required for the development of the two embryonic lineages. CRF1-mediated signalling is required for suspensor PCD in tobacco. Auxin produced in the micropylar seed coat is transported apically via the suspensor, and is necessary for the patterning of the embryo proper in Arabidopsis (bottom right panel). ALE1, ABNORMAL LEAF SHAPE1; CRF1, CYS REGULATIVE FACTOR 1; GSO1/2, GASSHO1/2; i.i., inner integument; o.i., outer integument; PCD, programmed cell death; PRC2, Polycomb Repressive Complex 2; TWS1, TWISTED SEED1.

Fitting with the notion that the endosperm is necessary for establishing dehydration responses in the embryo, the endosperm has been shown to be a decisive factor in the deposition of the embryonic cuticle (Fig. 2, bottom left panel). This cuticle is *de novo* deposited during embryogenesis and is necessary for water loss avoidance and for general plant development (Ingram and Nawrath, 2017). Two receptor kinases in Arabidopsis, called GASSHO1 (GSO1) and GSO2, are expressed in the embryo and necessary for cuticle formation (Tsuwamoto *et al*., 2008; Creff *et al*., 2019). The corresponding mutant embryos show developmental abnormalities, as well as increased epidermis permeability (Tsuwamoto *et al*., 2008; Creff *et al*., 2019). Another component of the GSO1/2 pathway is the subtilisin-like serine protease, ABNORMAL LEAF SHAPE1 (ALE1) (Xing *et al*., 2013; San-Bento *et al*., 2014). However, unlike GSO1/2, ALE1 is not expressed in the embryo, but rather in the endosperm (Tanaka *et al*., 2001), but the *ale1* mutant phenocopies *gso1*/*2* (Creff *et al*., 2019). A small embryonic peptide, called TWISTED SEED1 (TWS1), was shown to be a substrate for ALE1 and a ligand for GSO1/2 (Doll *et al*., 2020b). According to this model, TWS1 is produced by the embryo and exported to the endosperm, where it is cleaved by ALE1; if there are gaps in the embryonic cuticle, the cleaved TWS1 moves back to the embryo, where it is perceived by GSO1/2, instructing the embryo to actively deposit cuticle; once the cuticle is fully established, TWS1 can no longer move between seed compartments and this communication ceases (Doll *et al*., 2020b).

Other factors originating in the endosperm are thought to impact on embryogenesis. For example, the embryo surrounding region (ESR) of the maize endosperm plays an important role in mediating auxin homeostasis. No auxin activity was reported in the ESR using the *DR5* auxin reporter (Doll *et al*., 2017). Also, the auxin efflux carriers, ZmPINs, localize in the endomembrane system instead of plasma membrane in the ESR leading to the suggestion that ESR acts as a hormonal buffer zone preventing polar auxin transport from the endosperm to the embryo at the early development stages (Doll *et al*., 2017). As the embryo grows and exits this region, it becomes exposed to hormones, like auxins, in the non-ESR, which coincides with the first hormonal response in the apical regions of the embryo followed by morphogenesis (Chen *et al*., 2014; Doll *et al*., 2017). Fitting with this, the auxin exporter ZmPIN1 is initially localized in the apical epidermal cells of the maize embryo (Forestan *et al*., 2010), suggesting that it could contribute to importing auxin originating in the endosperm into the embryo.

While the formation of an endosperm is critical for embryo viability, the opposite would not necessarily seem to be the case. In Arabidopsis, endosperms developing without a neighbouring embryo are morphologically similar to those from seeds derived from double fertilization, at least during early stages of development (Xiong *et al*., 2021). These experiments relied on a mutant called *kokopelli* (*kpl*), whose pollen often only carries one gamete, instead of two (Ron *et al*., 2010). This means that when this mutant is used as a father, a proportion of ovules are only fertilized once. This originates seeds that only contain an endosperm, but no embryo, or vice versa. Siblingless endosperms produced in this way proliferate, expand, and undergo timely cellularization, in a manner seemingly similar to those forming with a neighbouring embryo (Xiong *et al*., 2021). Naturally, because in this situation there is no embryo to consume the endosperm, the latter does not break down, and persists in the mature seed (Xiong *et al*., 2021). Similarly, in maize, the endosperm still forms an embryo-pocket in embryo defective (*emb*) mutants, even if no embryo is present (Clark and Sheridan, 1991; Mimura *et al*., 2018). The formation of this cavity, which normally accommodates the embryo, requires the expression of an RKD-type RWP-RK transcription factor-encoding gene, *Shohai1* (*Shai1*), in the endosperm of *emb* mutants of maize (Mimura *et al*., 2018). This means that, to some degree, the morphological development of the endosperm is autonomous and not dependent on signals originating in the embryo. A recent study leveraged *kpl* mutants to show that the central cell fertilization is necessary for degradation of the callose plug at the terminal part of the phloem and transfer of nutrients from the maternal plant to the ovule in both Arabidopsis and rice (Liu *et al*., 2025). By contrast, the egg cell fertilization does not trigger gate opening in the absence of central cell fertilization. This explains the role of endosperm in nutrient loading from the maternal tissues, irrespective of the presence of the embryo.

However, another study in the early Arabidopsis endosperm shows that the absence of an embryo does, in fact, impact gene expression (Zhang *et al*., 2023). These authors also resorted to the *kpl* mutant as a father, and observed that there are measurable and significant changes in transcriptomes between endosperms developing with or without a neighbouring embryo (Zhang *et al*., 2023). Deregulated genes included those encoding transcription factors, some of which are known to have important functions during endosperm development (Zhang *et al*., 2023). Consistent with this, the absence of an embryo in maize seeds also leads to changes in expression of genes associated with the endosperm adjacent to scutellum (Doll *et al*., 2020a). This indicates that the embryo does indeed affect the development of the endosperm, even if the morphology of the early endosperm is not obviously changed. Importantly, such effects on endosperm formation seem to be dependent on the presence or absence of an embryo, rather than on its genotype. Experiments in maize, where two genetically distinct fathers were used to co-pollinate the same mother, yield a small percentage of heterofertilized seeds, that is, where the embryo and the endosperm are derived from different fathers, as opposed to homofertilized seeds, where the embryo and endosperm share both parents. Interestingly, in those heterofertilized seeds, the mature maize kernels yield smaller embryos, when compared with those resulting from homofertilization (Wu *et al*., 2013). The opposite, however, is not observed. That is, the endosperm weight of heterofertilized and homofertilized seeds is similar (Wu *et al*., 2013). Overall, this means that the presence of an embryo does likely contribute to endosperm development, but the actual genetic composition of the embryo and its relatedness to the endosperm seemingly play a more limited role.

## The endosperm and the seed coat develop in a coordinated manner

The seed coat is the only component of the seed that is not fertilized, as it develops from the ovule integuments. Nevertheless, double fertilization triggers the growth and differentiation of the seed coat (Haughn and Chaudhury, 2005). This means that the integuments must receive some sort of signals from the fertilized gametes, instructing them to initiate seed coat formation. Several pieces of data from Arabidopsis indicate that it is the endosperm, not the embryo, which is the source of those signals. First, genetic ablation of the endosperm by tissue-specific expression of the diphtheria toxin A chain resulted in cessation of seed growth, meaning that without an endosperm, the seed coat does not grow (Weijers *et al*., 2003). Conversely, mutations in *FERTILIZATION-INDEPENDENT SEED* (*FIS*) genes, which result in the formation of endosperms without fertilization, but not embryos, also lead to the formation of seed coats (Ingouff *et al*., 2006). Finally, single fertilizations of the embryo sac confirmed those observations (Roszak and Köhler, 2011). These experiments again resorted to the use of the *kpl* Arabidopsis mutant, to produce seeds containing only an embryo or only an endosperm. Strikingly, only those seeds containing an endosperm could develop a seed coat (Roszak and Köhler, 2011). The seeds that contained an embryo but no endosperm did not grow and the integuments collapsed after a few days (Roszak and Köhler, 2011). This means that the Arabidopsis endosperm is the source of a signal that triggers the development of the ovule integuments into a seed coat.

Because the development of the seed coat is triggered by the fertilization of the central cell, it is likely that the initiation signals are encoded by the paternal genome. Nonetheless, pollen tube content is sufficient to initiate ovule enlargement and seed coat development in Arabidopsis (Kasahara *et al*., 2016), meaning that seed coat initiation likely relies on both fertilization and on factors delivered by the pollen tube. The endosperm is a site for a phenomenon known as genomic imprinting, whereby certain genes are preferentially expressed from the maternal or the paternal alleles (reviewed in Batista and Köhler, 2020). Among those genes that are paternally expressed in the endosperms of Arabidopsis are those encoding auxin biosynthesis genes, *YUCCAs* (*YUC*) and *TRYPTOPHAN AMINOTRANSFERASE*/*TAA-RELATED* (*TAA*/*TARs*) (Gehring *et al*., 2011; Hsieh *et al*., 2011; Wolff *et al*., 2011; Figueiredo *et al*., 2015), and, indeed, post-fertilization auxin activity in the integuments can be detected using the *DR5* auxin reporter (Dorcey *et al*., 2009). This led to the hypothesis that auxin produced after fertilization in the endosperm could couple seed coat development to fertilization. Indeed, when auxin biosynthesis genes are ectopically expressed in the unfertilized central cell, the seed coat starts developing as though fertilization had taken place (Figueiredo *et al*., 2016). Moreover, if auxin is exogenously applied to unfertilized ovules, auxin responses are triggered and the seed coat starts forming without fertilization (Dorcey *et al*., 2009; Figueiredo *et al*., 2016). In Arabidopsis, this phenotype can be easily scored by the characteristic accumulation of proanthocyanidins in the integument endothelium, which is a marker for seed coat formation (Debeaujon *et al*., 2003; Figueiredo *et al*., 2016). Interestingly, post-fertilization auxin accumulation seems to be a characteristic feature of endosperm development in angiosperms (Florez-Rueda *et al*., 2024a, b, Preprint), and exogenous applications of auxin lead to fertilization-independent development of the sporophytic tissues of the seeds in early diverging angiosperms (Florez-Rueda *et al*., 2024b, Preprint), suggesting that the mechanisms coupling fertilization to seed coat formation may be conserved to some degree among the flowering plants. Another hormone whose activity is detected in the integuments post-fertilization is gibberellin (GA). Although GA applications also trigger seed coat formation (Figueiredo *et al*., 2016), GA is seemingly downstream of endosperm-derived auxin during seed coat development (Dorcey *et al*., 2009; Figueiredo *et al*., 2016).

The observations that paternal instructions are necessary for the seed coat to form also led to the hypothesis that the mother actively suppresses seed coat formation in case the ovule is not fertilized. Indeed, the formation of the seed coat in Arabidopsis is suppressed by the deposition of repressive H3K27me3 marks by the Polycomb Repressive Complex 2 (PRC2) (Roszak and Köhler, 2011). Two complexes, named VERNALIZATION (VRN)–PRC2 and EMBRYONIC FLOWER (EMF)–PRC2, were shown to redundantly prevent fertilization-independent seed coat formation (Roszak and Köhler, 2011). Consistent with this, mutations in components of these complexes lead to ovules producing seed coats without having been fertilized (Roszak and Köhler, 2011; Figueiredo *et al*., 2016). This also means that these PRC2s, as well as the epigenetic marks that they deposit, need to be removed after fertilization, so that the seed coat can develop. The removal of the PRC2s seems to be determined by endosperm-derived auxin, as it coincides with the down-regulation of genes encoding PRC2 subunits either by fertilization, or by exogenous applications of auxins (Fig. 2, upper left panel) (Figueiredo *et al*., 2016). Nevertheless, even if the PRC2s are removed, the H3K27me3 marks that they deposited are stable in the non-dividing seed coat cells. These repressive epigenetic marks are then removed from the integuments via the activity of histone demethylases of the JUMONJI-type (JMJ) (Pankaj *et al*., 2023, Preprint). If *JMJs* are mutated, the seed coat cells cannot elongate properly, nor do they accumulate the characteristic proanthocyanidins in the endothelium (Pankaj *et al*., 2023, Preprint). Moreover, this removal of H3K27me3 seems to be under the control of the Brassinosteroid (BR) receptor BRASSINOSTEROID RESISTANT 1 (BRI1), as mutations in *BRI1* lead to seed coat defects and hyperaccumulation of H3K27me3 (Pankaj *et al*., 2023, Preprint). In conclusion, seed coat formation relies on auxin derived from the endosperm, which removes PRC2s, and subsequent removal of the epigenetic marks via a concerted action of BRI1 signalling and JMJ histone demethylases.

In addition to the chemical signals that trigger seed coat formation, the growth of the seed coat is also dependent on the physical pressure exerted on it by the expanding endosperm and embryo. The cell wall that is at the interface between the inner and outer integuments of the Arabidopsis ovule is particularly sensitive to internal pressure (Fig. 2, upper right panel): in *haiku2* (*iku2*) mutants, in which the endosperm expansion is reduced, this cell layer is much thinner than in the wild type (WT) (Creff *et al*., 2015). The thickening of this cell wall correlates with the strength of expression of the *ELA1* gene, which encodes a cytochrome P450 monooxygenase (Creff *et al*., 2015). This gene is expressed in the innermost layer of the outer integument of the seed coat, and its expression is induced by physical pressure (Creff *et al*., 2015). Interestingly, ELA1 is a known regulator of GA metabolism (Zhang *et al*., 2011). Therefore, it is likely that increased expression of ELA1 induces seed coat expansion by modulating GA metabolism, as a response to pressure from the inner seed compartments. Because GA activity is also known to be downstream of auxin in the integuments (Dorcey *et al*., 2009; Figueiredo *et al*., 2016), it is likely that both the removal of PRC2 and activation of ELA1 signalling converge on GA responses, as a general mechanism to drive seed coat growth.

This role of the endosperm in promoting seed coat expansion is in fact well-established in the Arabidopsis literature. For instance, mutations in components of the HAIKU (IKU) pathway, such as the aforementioned IKU2, but also IKU1, MINISEED3 (MINI3) and SHORT HYPOCOTYL UNDER BLUE1 (SHB1), result in less proliferative endosperms (Garcia *et al*., 2003; Luo *et al*., 2005; Zhou *et al*., 2009; Wang *et al*., 2010). As a consequence, the corresponding seed coats also grow less, when compared with their WT counterparts (Garcia *et al*., 2003; Luo *et al*., 2005; Zhou *et al*., 2009; Wang *et al*., 2010). Because the IKU pathway is specific to the endosperm, the effect of these mutations on seed coat formation is indirect. In this pathway, the SYG1 family protein SHB1 activates the expression of WRKY family transcription factor *MINI3* and a leucine-rich receptor kinase encoding gene, *IKU2* (Zhou *et al*., 2009). Consequently, the *shb1-D* gain-of-function mutation, which leads to increased *MINI3* and *IKU2* expression in the endosperm, results in much larger seeds when compared with the WT (Zhou *et al*., 2009). This large seed phenotype often coincides with a longer coenocytic phase of the endosperm, whose final size is thought to be controlled by F-actin filaments (Ali *et al*., 2023). The mechanosensitive seed coat then adjusts its growth to this increased expansion of the zygotic products. Surprisingly, however, pressure originating in the endosperm can actually have contrasting effects on seed coat expansion: while the endosperm growth has an initial promotive effect on the seed coat, as discussed above, by promoting the mechanosensitive stiffening of the cell walls, it gradually generates more resistance for seed coat growth (Creff *et al*., 2023). To demonstrate this the authors leveraged the *iku2* mutants that exhibit small seed size due to higher endosperm pressure. Interestingly, this phenotype can be partially complemented by altering the differentiation of the testa cell wall with impaired outer-integument identity *ap2* mutants (Creff *et al*., 2023).

The effect of the zygotic products in shaping the growth of the seed coat is not surprising, as the seed coat has to adjust its growth to accommodate the endosperm and the embryo. However, this communication is in fact bi-directional. That is, the expansion of the seed coat also has a measurable effect on endosperm development. This was initially observed in Arabidopsis mutants where the growth of the seed coat is compromised. For instance, mutations in the gene *TRANSPARENT TESTA GLABRA2* (*TTG2*) suppress cell elongation in the seed coat (Garcia *et al*., 2005). As a consequence, not only are the resulting seed coats smaller, but the endosperm cavity in the mutant seeds is largely reduced (Garcia *et al*., 2005). Conversely, mutants in *APETALA2* (*AP2*), where the seed coat cells elongate more than in the WT, produce larger endosperms (Ohto *et al*., 2009). In this case, the increased size of the endosperm correlates with a delay in the timing of endosperm cellularization and, thus, an extended period of coenocytic growth (Ohto *et al*., 2009). These observations indicate that the endosperm also coordinates its expansion to the growth of the seed coat and, thus, should receive signals that originate in the sporophytic tissues. The BR hormones have been implicated in the communication between these two tissues. BR biosynthesis and signalling produce seeds whose coats are smaller and differently shaped, when compared with those of WT seeds (Jiang *et al*., 2013). However, such mutants also produce endosperms that proliferate more slowly, even when a WT pollen donor is used (Lima *et al*., 2024). This means that BR activity in the seed coat determines the proliferation rate of the endosperm. Surprisingly, these effects are not due to chemical signalling between seed coat and endosperm, but are rather linked to physical cues. This is because unrelated mutants that restrict seed coat expansion also show a slowdown of endosperm proliferation: the stronger a given mutation restricts seed coat expansion, the less the endosperm proliferates (Lima *et al*., 2024). Consistently, seeds that develop under the physical pressure of glass slides also show reduced endosperm proliferation (Lima *et al*., 2024). Surprisingly, the physical restriction that the seed coat imposes on the endosperm does not seem to affect the duration of the coenocytic phase, because the timing of endosperm cellularization remains unaffected (Lima *et al*., 2024). Therefore, the role of the seed coat in restricting endosperm proliferation seems to be uncoupled from the timing of its cellularization.

## Early embryo development requires signals originating in the seed coat

As alluded to above, the early Arabidopsis embryo can develop up to an early globular stage without the presence of an endosperm. However, factors originating in the sporophytic tissues, namely the seed coat, are seemingly necessary for early embryogenesis. After fertilization, the embryo undergoes an asymmetric cell division, which creates a small apical and a larger basal cell (reviewed in Wendrich and Weijers, 2013). The former will give rise to the embryo proper, and the latter will give rise to the suspensor (Wendrich and Weijers, 2013). Importantly, the patterning of the embryo proper requires a flow of auxin coming from the basal cell (Robert *et al*., 2013). However, the source of this auxin remained unclear until it was shown that the micropylar integuments can provide auxin to the proembryo via the suspensor (Fig. 2, bottom right panel) (Robert *et al*., 2018). Consistent with this idea, Arabidopsis embryos developing surrounded by seed coats that are compromised in auxin biosynthesis show patterning defects (Robert *et al*., 2018). Moreover, these maternal tissues are also necessary for the fate of the suspensor cells. The suspensor is a transient tissue and it eventually undergoes programmed cell death (PCD) and degenerates (reviewed in Peng and Sun, 2018). Interestingly, signals for suspensor PCD seem to originate in the sporophytic maternal tissues in tobacco (*Nicotiana tabacum*) (Shi *et al*., 2019). Suspensor PCD is dependent on a cystatin-cathepsin protease module called NtCYS–NtCP14 (Zhao *et al*., 2013), whose expression is regulated by a DELLA protein, NtCYS REGULATIVE FACTOR 1 (NtCRF1) (Fig. 2, bottom right panel) (Shi *et al*., 2019). DELLA proteins are well-established regulators of gibberellin (GA) responses (reviewed in Ito *et al*., 2018). And, indeed, ectopic production of GA in the seed coat endothelium leads to a degradation of NtCRF1 and a trigger of premature PCD in the suspensor lineage (Shi *et al*., 2019). These observations mean that the sporophytic tissues contribute signals that modulate the development of the two embryonic lineages, embryo proper and suspensor.

The development of the seed coat can also indirectly impact embryo formation via its effects on endosperm development. Because the growth of the seed coat dictates how much the endosperm can grow, this also limits how much resources the embryo can consume. For example, the *apetala2* mutation (*ap2*) in Arabidopsis leads to an enlarged seed coat (Ohto *et al*., 2005). As discussed above, the consequence of such maternal effects is a promotion of endosperm expansion. However, the indirect consequence is that the consumption of those larger endosperms leads to embryos that are much larger than those growing surrounded by WT seed coats (Ohto *et al*., 2009). In fact, *ap2* seeds seem to be stronger sinks for hexoses (Ohto *et al*., 2005), which are known to accumulate in the endosperm (Weber *et al*., 1997; Lafon-Placette and Köhler, 2014). This accumulation of metabolites is likely linked to the extended coenocytic phase of *ap2* endosperms (Ohto *et al*., 2009). Thus, the degree to which the seed coat determines how much the endosperm grows has unavoidable impacts on the embryo development.

To our knowledge, no role of the embryo in seed coat development has been *de facto* established in model species, suggesting that the endosperm is the sole source of non-cell autonomous signals to the seed coat, which originate in the fertilized products. Nevertheless, the expansion of the embryo likely contributes to the internal physical pressure exerted on the seed coat (Creff *et al*., 2015). Naturally, we cannot exclude that there are unidentified signals originating in the embryo that do affect seed coat formation. However, because embryos always have to develop accompanied by an endosperm, it becomes very difficult to isolate factors originating in the embryo alone.

## Conserved players with diversified roles in monocots and dicots

While the key developmental processes and molecular regulators are largely conserved in dicots and monocots, the functional diversification of conserved players shapes the distinct functional and structural characteristics of their seeds. For example, LEAFY COTYLEDON 1 (LEC1), considered one of the master regulators of seed development, is present in both dicots and monocots. *LEC1* is expressed in both embryo and endosperm in Arabidopsis and is essential for embryo maturation (Song *et al*., 2021). In the absence of endosperm-expressed *LEC1*, embryo development arrests even in the presence of a functional allele of embryo-expressed *LEC1*, indicating that endosperm-expressed *LEC1* is necessary and sufficient for embryo maturation in Arabidopsis and regulates embryo maturation in a non-autonomous manner (Song *et al*., 2021). Rice has two homologs of *LEC1*, *OsNF-YB7* and *OsNF-YB9*, which exhibit distinct spatiotemporal expression patterns: *OsNF-YB7* is expressed in embryos, while *OsNF-YB9* is mainly detected in the endosperm (Zhiguo *et al*., 2018). Interestingly, heterologous expression of either of them driven by *LEC1* native promoter could complement the *lec1-1* defects in Arabidopsis (Niu *et al*., 2021). While *OsNF-YB7* mutants in rice were lethal, indicating its role in embryo development, the loss of function of *OsNF-YB9* led to elongated grains and chalky endosperm (Niu *et al*., 2021). Since chalkiness is a condition associated only with monocot seeds due to defects in starch accumulation in the endosperm, the mutant phenotypes of *LEC1* orthologs in rice and Arabidopsis exemplify the functional divergence of shared genes between dicots and monocots to account for different morphologies and ecological adaptations.

Another interesting example is the maize *Shohai1* (*Shai1*) gene, which encodes an RKD-type RWP-RK transcription factor that regulates embryonic patterning. The *shai1* mutants develop embryos of variable sizes and shapes and, as alluded to above, *Shai1* was shown to be required for the autonomous formation of embryo pockets in the endosperm (Mimura *et al*., 2018). Expression of *shai1* in endosperm could partially rescue the embryo mutant phenotype, indicating its non-cell autonomous signalling from the endosperm helps embryo growth (Mimura *et al*., 2018). *Shai1* is also required for endosperm development, as abnormal growth and pigmentation in aleurone were observed in the *shai* mutant (Mimura *et al*., 2018). *AtRKD5* is the closest orthologue of *Shai1* in Arabidopsis. Loss of function of *AtRKD5* does not have a discernible phenotype and, based on transcriptional studies, it was implicated in regulating the ethylene pathway during ovule development (Tedeschi *et al*., 2017). These results confirm that specific roles of the *Shai1*/*AtRKD5* gene have diversified during the evolutionary divergence of dicots and monocots.

## Implications of the seed coat, embryo, and endosperm interactions for agronomic traits

**Fig. 3.**
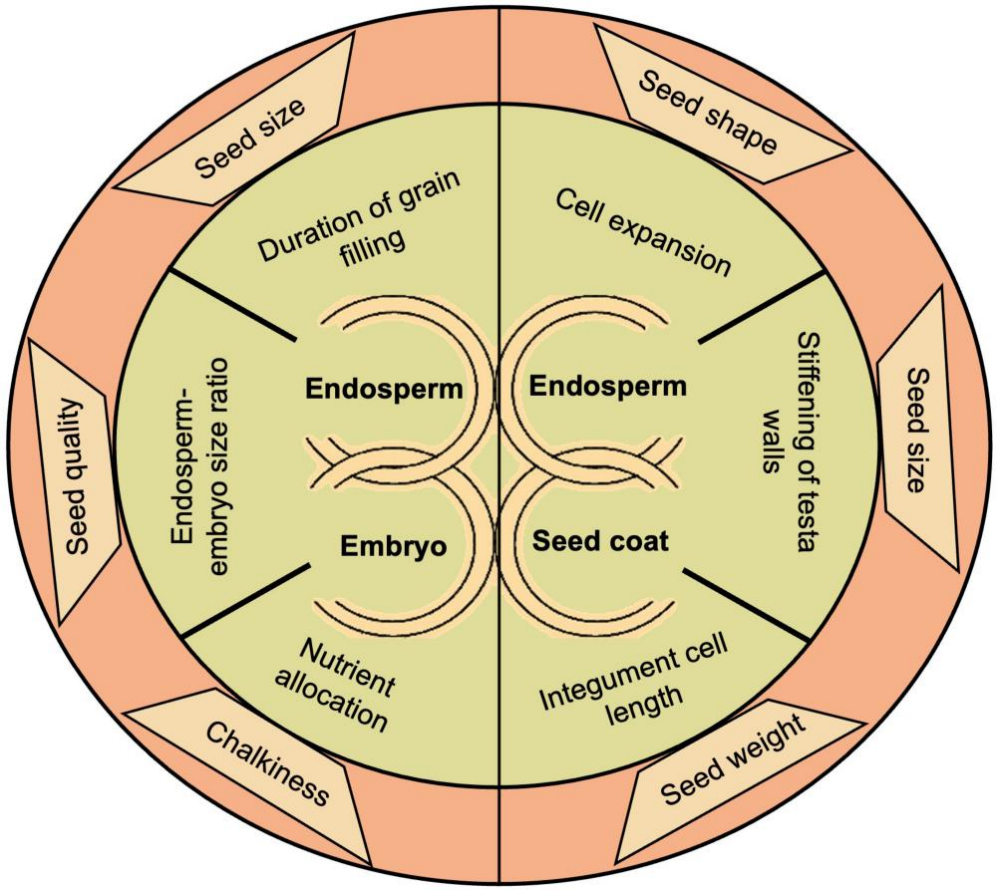
Key biological processes and agronomic seed traits regulated by endosperm–embryo–seed coat interactions. The inner circle presents the biological processes directly impacted by the interactions between endosperm and embryo, and endosperm and seed coat, which in turn determine seed agronomic traits mentioned in the outer circle.

The interactions between the seed coat, embryo, and endosperm play a pivotal role in regulating seed size, shape, and composition. Though the optimal seed size and shape can vary with the plant species and specific management practices, larger seed size often significantly contributes to improved agronomic performance and enhanced economic returns. For example, increased seed size has been associated with improved germination, seed vigour and physiological characteristics in Arabidopsis and soybean (Krzyszton *et al*., 2024; Wang *et al*., 2025). In some crops, seed size is a direct determinant of market value. For example, in rice, the grain size and shape largely affect consumers’ choice and hence the price, with some varieties carrying a premium perception due to their specific sensory and culinary attributes (Boccaccini *et al*., 2024). Therefore, by targeted engineering of the embryo, endosperm, and seed coat interactions, plant scientists can develop improved crop varieties with higher yields, nutritional attributes, and economic value. Figure 3 highlights the impact of endosperm–embryo interaction on seed size and quality and the contributions of endosperm–seed coat interactions on seed size, shape, and weight as highlighted by specific examples in the sections below.

### Bidirectional communication between endosperm and seed coat regulates seed size, shape, and weight

The communication between endosperm and seed coat plays a vital role in regulating seed size. Several zygotic and sporophytic maternal tissue-expressed genes are known to modulate seed size by regulating the elongation of integument cells and the timing of endosperm cellularization (Garcia *et al*., 2005). We alluded to some of these interactions in the sections above. One additional example is that mediated by the cytochrome P450 KLUH (KLU)/CYP78A5, which regulates seed size in Arabidopsis, although its expression is exclusively detected at the base of the nucellus and inner integument (Adamski *et al*., 2009). Loss-of-function and overexpression studies confirmed its role in regulating seed size and weight by regulating integument cell number. Interestingly, *KLU* is only required in sporophytic tissues to promote seed growth, suggesting that a KLU-dependent mobile growth signal crosses the inner and outer integuments to stimulate endosperm growth (Adamski *et al*., 2009). Although the nature of this mobile signal still remains elusive, a model suggesting the transport of CYP78A-derived growth signal from endoplasmic reticulum to the Golgi was recently proposed (Zhou *et al*., 2023). This was discovered while working on rice *small grain 4* (*smg4*) mutant. *SMG4* encodes a multidrug and toxic compound extrusion (MATE) transporter, which transports this signal to the Golgi by Coat Protein Complex Ⅱ-mediated vesicle transport, thereby promoting cell expansion and proliferation (Zhou *et al*., 2023).

Similarly, loss of function of *TRANSPARENT TESTA GLABRA2* (*TTG2*), a WRKY transcription factor in Arabidopsis, led to small seeds due to reduced elongation of cells of integuments and precocious cellularization of the endosperm (Garcia *et al*., 2005). Although *TTG2* expression is mainly localized in the endothelium, the innermost layer of integument, the impact of this gene on endosperm cellularization confirms the role of maternal sporophytic factors in regulating endosperm development and, hence, seed size. Conversely, endosperm-derived VQ-motif containing protein IKU1 regulates cell dimension in integuments in addition to endosperm proliferation (Garcia *et al*., 2003). Interestingly, *TTG2* is mainly implicated in the flavonoid biosynthesis pathway. Flavonoids have earlier been shown to regulate *PIN-FORMED* (*PIN*) expression and protein localization by altering local auxin concentration and cellular trafficking (Peer *et al*., 2004). Later they were shown to modulate auxin transport by directly binding to the ATP-binding cassette subfamily B (ABCB) transporters, also known as P-glycoproteins, or modulating the activity of regulatory kinases and phosphatases associated with auxin transport (Peer and Murphy, 2007). Although the mechanistic details of the impact of flavonoids in regulating seed size remain unclear, their emerging role as auxin transport regulators suggests that morphogenic properties of auxin may be responsible for the observed phenotype (Doughty *et al*., 2014). The role of auxins in this bidirectional dialogue to determine seed size has been recently reviewed elsewhere (Cao *et al*., 2020; Liu *et al*., 2023).

BRs have also been receiving increasing attention as modulators of seed size and overall plant yield (Lima and Figueiredo, 2024; Yang *et al*., 2024). Expression profiling of seed size-related genes in BR deficient mutant *det2* of Arabidopsis in response to BR and brassinazole (which blocks BR biosynthesis) indicated that BRs activate expression of positive regulators of seed size, including *SHB1*, *MINI3*, and *IKU2*, but represses expression of negative regulators including *APETALA2* and *AUXIN RESPONSE FACTOR2* (Jiang *et al*., 2013). This highlights a potential role of BRs in transcriptional regulation of key genes coordinating seed coat and endosperm development to regulate seed size. Furthermore, as we also discussed above, the developmental control of the turgor pressure from the developing endosperm regulates seed growth and size. Given the roles auxins and BRs play in maintaining endosperm growth and pressure, as well as the structural integrity of seed coat cell walls, manipulating the levels of BRs and auxins can be an important strategy to regulate seed size in crop plants.

Another interesting example comes from the role of the ERECTA (ER)–mitogen-activated protein kinase 4/5 (MKK4/5)–mitogen-activated protein kinase 3/6 (MPK3/6)–DA1–Ubiquitin Specific Protease 15 (UBP15) signalling cascade in determining seed size by regulating outer integument cell proliferation in Arabidopsis (Wu *et al*., 2022). The receptor-like kinase ER transduces the signal for MKK4/5–MPK3/6-mediated phosphorylation and inactivation of the ubiquitin receptor DA1 (DA means big in Chinese), causing the enhanced accumulation of UBP15 and consequent cell proliferation in outer integument cells. Interestingly, this pathway seems conserved in crop plants as well. Down-regulation of *BnDA1*, by overexpressing the negative interfering mutant of Arabidopsis *DA1*, led to enhanced seed size and weight in *Brassica napus* (Wang *et al*., 2017). Similarly, overexpression of mutated *ZmDA1* in maize resulted in improved kernel yield, seed size, and starch content (Xie *et al*., 2018). The rice homolog of Arabidopsis *DA1*, *OsDA1*, has recently been implicated in modulating grain size and shape by regulating cell enlargement. Overexpression of *OsDA1* led to enlarged seeds with increased width, while loss of function resulted in increased grain length/width ratio (Li *et al*., 2024). These studies underline the conserved role of *DA1* as a promising target for engineering seed shape and size in crop plants.

### Embryo-endosperm size ratio and nutrient allocation between them determine the size and overall quality of seeds

Although a delicate balance between embryo and endosperm is maintained during seed development, with embryos typically comprising only 2–3% of kernel weight in rice, larger embryo size has been linked to enhanced nutritional value. Several mutants, such as *endospermless*, *embryoless*, *reduced embryo*, *giant embryo*, *super-giant embryo*, and *large embryo*, have been identified in rice with distinct embryo and endosperm-size related phenotypes (Hong *et al*., 1996; Koh *et al*., 1996; Lee *et al*., 2019). Among these, *giant embryo* mutants have been actively used for improving the nutritional value of rice with enhanced proteins, lipids, vitamins, minerals, essential amino acids, and bioactive compounds compared with WT cultivars with normal embryo size. The black waxy rice developed with *giant embryo* produces high quality nutritional compounds including γ-aminobutyric acid, amylopectin, and bioactive anthocyanin in rice bran (Zhang *et al*., 2005; Jeng *et al*., 2012; Kim *et al*., 2013). In another study, rice mutants *mge8*, *12*, *13*, and *14* with larger embryo were reported to contain higher triacylglycerol levels than the WT seeds, suggesting that larger embryo size can also be used for breeding rice varieties with improved bran oil content (Sakata *et al*., 2016). In an independent study in rice, Xu *et al*. (2015) identified a dominant mutant, *big grain 2* (*bg2-D*), by screening an enhancer-trapping population. *BG2* encodes a cytochrome P450 protein, OsCYP78A13, which was earlier identified as GIANT EMBRYO (Nagasawa *et al*., 2013; Yang *et al*., 2013). The phenotypic analysis of dominant mutant and overexpression lines for this gene confirmed its role in regulating overall seed size and fatty acid content in addition to the earlier reported role in regulating embryo-endosperm size ratio. Overexpression of *BG2* and its paralogue *Grain Length 3.2* (*GL3.2*) led to increased seed size in rice. Analysis of sequence diversity in 1529 rice varieties suggested that variation in the coding region of *CYP7813* is responsible for variation in grain yield in *indica* cultivars of rice (Xu *et al*., 2015).

Manipulating genes associated with nutrient partitioning between the embryo and endosperm also seems a promising route for tailoring seed quality for specific agricultural and nutritional goals. For example, the rice gene *OsYSL9*, encoding an iron-chelate transporter, is expressed in the scutellum of the embryo and endosperm cells surrounding the embryo in rice (Senoura *et al*., 2017). Down-regulation of *OsYSL9* led to reduced iron content in the embryos and higher accumulation in endosperm, confirming its role in maintaining intercompartmental Fe homeostasis (Senoura *et al*., 2017). Since rice is mostly consumed after polishing, where the outermost seed coat and embryo are removed, the higher Fe content in the endosperm is desirable to tackle iron deficiency anaemia in developing countries. Similarly, Viviparous 1 (VP1), a B3 family transcription factor in maize, coordinates embryo–endosperm interaction and nutrient allocation from endosperm to embryo (Zheng *et al*., 2019). The loss of function of *VP1* suppressed the accumulation of many proteins in embryos by hampering scutellum development and its ability to incorporate endosperm-transferred nutrients. Although *VP1* regulates several other aspects of seed development as well, including maturation and dormancy, future studies will clarify if it can be targeted for genetic improvement of protein quality in maize.

### Bidirectional communication between endosperm and embryo regulates chalkiness in rice seeds

Chalkiness in rice refers to the appearance and texture of rice grains marked by an opaque portion found in the otherwise translucent ventral and central part of the endosperm due to incomplete accumulation of starch and protein during grain filling. Chalkiness negatively impacts the appearance, milling, eating, and cooking quality of rice and, hence, is highly undesirable. Lin *et al*. (2014) identified a notched-belly mutant with a high percentage of white belly, only in the basal part of the endosperm near the embryo while the upper part is translucent (Lin *et al*., 2014). Comparative proteomic and transcriptomic analysis of these two portions of the endosperm indicated the role of the embryo in negatively affecting the storage of total protein, amino acids, and minerals in the chalky endosperm (Lin *et al*., 2016, 2017). Integrated analysis of transcriptome and metabolites from WT and *notched belly* (*NB*) mutant of rice showed that the extraction of nutrients by the embryo from the lower part of the endosperm, adjacent to embryo, leads to a metabolic shift from synthetic storage to secondary pathways and hence impaired grain filling and chalkiness (Tao *et al*., 2022b). The authors further suggest that the embryo acts as an internal timer for endosperm maturation by mediating the trehalose-6-phosphate (T6P)–SNF1-RELATED PROTEIN KINASE 1 (SnRK1) pathway in rice (Tao *et al*., 2022a). During early stages (5–10 d after fertilization), the embryo exerts a negative effect on sucrose levels, accompanied by a decline in T6P levels. This abolishes the inhibition of SnRK1 leading to higher catabolic activity. Between 20 and 25 d after fertilization, the T6P–SnRK1 signalling shows the opposite trend promoting starch biosynthesis. In agreement with this, authors further proposed a dragging effect of the embryo on the developmental transition of the endosperm in the *NB* mutant as duration of endosperm filling was prolonged in the lower part of endosperm, which is in closer proximity to the embryo compared than the upper portion (Tao *et al*., 2022a). To date, the *NB* gene product and its molecular function remain to be characterized.

An endosperm-expressed *LEC1* orthologue in rice, *OsNF-YB9*, has also been implicated in regulating grain length and endosperm chalkiness by regulating the expression of genes related to starch biosynthesis. Loss of function of *OsNF-YB9* or its interaction partner *Sucrose synthase Protein Kinase* (*SPK*) led to a higher chalkiness ratio in rice (Niu *et al*., 2021). While endosperm-expressed *LEC1* in Arabidopsis is only implicated in the regulation of embryo development, rice orthologues of *LEC1*, *OsNF-YB7*, and *NF-YB-9* seem to have undergone subfunctionalization with *NF-YB7* regulating embryo development and *OsNF-YB9* regulating seed size and grain filling (Zhiguo *et al*., 2018). These results also demonstrate the functional diversification of conserved players in dicots and monocots to account for morphological and physiological differences in seed development.

## Challenges in deciphering communication between seed coat, endosperm, and embryo

Although synchronization between the three generations within the developing seeds piqued the curiosity of many researchers in the past decades, limitations of current technology and the multilevel complexity of the interactions between them continue to present a major challenge in deciphering the communication between embryo, endosperm, and seed coat. First of all, these interactions are under strict spatiotemporal control in the context of development as well as the chronology of events during seed development. Secondly, the tissues at the interface of embryo and endosperm are transcriptionally distinct with specialized roles in nutrient transport and regulation (Doll *et al*., 2020a). The idea of tracking their development and interaction with adjacent tissues is still elusive due to the continuous nature of these tissues and variability across different species. For example, unlike Arabidopsis, cereal crops have highly specialized basal endosperm transfer layers at the interface with maternal tissues, distinct from the embryo–endosperm boundary. It is a highly dynamic and regulated tissue, central to determining the final size and composition of the seed by regulating the transfer of nutrients from maternal tissues to the developing seed. However, the anatomy of this maternal–filial interface is so variable in cereal crops that extrapolating the findings from one species to another is clearly not feasible without rigorous validations.

Furthermore, the non-autonomous regulation during seed development compounds the challenge of deciphering inter-tissue interactions by introducing indirect effects and long-range signalling that obscures direct cause-and-effect relationships. The role of small peptides in cell–cell communication during plant development is well demonstrated (Fukuda and Higashiyama, 2011). Typically, precursor peptides undergo proteolytic cleavage and post-translational modifications to give rise to biologically active peptides that are then recognized by membrane-bound receptor kinases to exert their functions. These peptides are ideal for short and long-distance communication and non-cell-autonomous regulation. Several peptides have been implicated in regulating seed development in a non-cell-autonomous manner (Ingram and Gutierrez-Marcos, 2015). One example that we mentioned above is the cysteine-rich peptides, like EMBRYO SURROUNDING FACTOR 1 (ESF1.1 to 1.3), which specifically accumulate in the central cell and embryo-surrounding endosperm cells of Arabidopsis before and after fertilization, respectively (Costa *et al*., 2014). Genetic studies showed that ESF1 peptides, along with a receptor-like kinase, SHORT SUSPENSOR, regulate suspensor elongation in a non-cell-autonomous manner through the YODA mitogen-activated protein kinase pathways (Costa *et al*., 2014). Another interesting example of the embryo–endosperm interaction mediated by small peptides comes from the discovery of *twisted seed1-1* (*tws1-1*) and *tws1-2* mutants, implicated in cuticle deposition on epidermal cells (Fiume *et al*., 2016). TWS1 is an embryo-derived peptide that undergoes tyrosine sulfation, and the sulfated peptide then diffuses to the surrounding endosperm, where it is cleaved by ALE1 to release the active peptide (Doll *et al*., 2020b; Royek *et al*., 2022). Notwithstanding these advances, although the repertoire of annotated peptides in plants is expanding, deciphering the peptide signalling networks is a complex and challenging task due to the low abundance, short half-life, dynamic nature, and high genetic redundancy of the peptide-encoding genes. The mode of transport of these mobile growth regulators across cell walls and membranes is barely understood. Further, the role of phytohormones in mediating non-cell-autonomous communication between different seed compartments to regulate seed size is also well established and has been reviewed elsewhere (Pankaj *et al*., 2025). Though the advances in imaging techniques, coupled with the use of conditional genetics and specific hormone agonists/antagonists, are beginning to unravel the intricacies and context-dependent roles of hormones in regulating seed development and inter-compartment interactions, the mechanistic details still remain elusive.

The timing of endosperm cellularization and, consequently, seed size also depends on the ratio of maternal and paternal genomes. Any deviations from the 2 maternal: 1 paternal genome ratio in the endosperm can have drastic effects on seed development (reviewed in Bente and Köhler, 2024). The excess of the paternal genome in 2*x*×4*x* crosses (the father being tetraploid) leads to delayed cellularization and hence larger seeds, while reciprocal maternal excess in 4*x*×2*x* crosses results in early cellularization, yielding small seeds in Arabidopsis (Scott *et al*., 1998), as well as in several agronomically important crop species, like rice (Sekine *et al*., 2013; Zhang *et al*., 2016), maize (Pennington *et al*., 2008), *Brassica oleracea* (Stoute *et al*., 2012), and many others. However, the positive effects of a father of higher ploidy on seed size is negated by high rates of seed abortion due to the endosperm not fully cellularizing (Bente and Köhler, 2024). Interestingly, genetic mutations in multiple pathways, including in paternally expressed imprinted genes, rescue these abortion phenotypes to some degree in Arabidopsis (Kradolfer *et al*., 2013; Wolff *et al*., 2015; Martinez *et al*., 2018; Satyaki and Gehring, 2019, Batista *et al*., 2019a, b; Huc *et al*., 2022; Xu *et al*., 2023; Zumajo-Cardona *et al*., 2023). One example of a pathway whose manipulation yields viable paternal-excess seeds is ABA: inhibition of ABA catabolism or external application of ABA suppressed the seed abortion phenotypes in Arabidopsis (Xu *et al*., 2023). Because the seeds obtained from such crosses are not tolerated in rice, maize, and many other crops, it would be interesting to see if manipulating ABA or other pathways can mitigate the effects of excess paternal dosage, opening new avenues for hybrid breeding programmes to enhance seed size.

Recent advances in high-resolution molecular profiling techniques have started to unveil a dynamic and delicate balance between paternal and maternal genomes in the endosperm. For example, single cell-based analyses of genomic imprinting in Arabidopsis demonstrated that imprinting is spatially and temporally heterogeneous in the endosperm (Picard *et al*., 2021), adding another layer of complexity to gene regulation in this tissue. Transcriptomic analysis of the endosperm-domain-enriched samples also demonstrated spatiotemporal regulation of imprinting during endosperm development (Van Ekelenburg *et al*., 2023). Ekelenburg and coworkers used fluorescence-activated nuclear sorting of fluorescent markers to capture parental-specific allelic expression from different development stages and domains of endosperm in Arabidopsis highlighting dynamic regulation of imprinting both in a spatial and a temporal context (Van Ekelenburg *et al*., 2023).

Evidently, to date, most of the studies elucidating genomic imprinting during seed development were either done in the dicot model Arabidopsis or in the monocot systems rice and maize. While Arabidopsis, being the most well-studied, is an obvious choice as an experimental system, the findings may not be universally translatable to outcrossing dicot crops. The predictability of parental conflicts from rice and maize is also questionable due to excessive artificial selection in these crops (Pires, 2014). Firstly, selective breeding does not align with the natural evolutionary interests of both parents and dramatically alters the selective pressures that shape these conflicts. For example, selection for larger seeds might disproportionately favour paternal interest for resource acquisition, exacerbating the conflict with the maternal strategy of more equitable resource distribution. Secondly, long-term artificial selection often leads to reduced genetic diversity that can inadvertently fix alleles that intensify parental conflicts or lead to loss of alleles involved in balancing parental demands. The evolutionary significance and long-term impacts of imprinting disruptions on plant development and associated agronomic traits remains to be investigated (Muthusamy *et al*., 2024). Overall, characterization of a more diverse range of plant systems from different taxonomic groups and with variable reproductive habits and ploidy levels would be desirable to avoid generalizations and understand species-specific modules and interactions governing seed development.

## Concluding remarks

Embryo, endosperm, and seed coat are all vital for successful seed development, and the communication and interactions between them are under strict chronological and developmental control. While the role of seed coat and endosperm on embryo development is indispensable, and the bidirectional dialogue between embryo and endosperm dictates the final seed phenotype, the influence of the embryo on surrounding tissues is not phenotypically discernible. Also, the strength and impact of these interactions is significantly variable across different developmental stages and crop species. Any variations in the developmental synchrony of these interactions within a species can have profound implications on seed agronomic traits. Manipulating these interactions for optimizing seed size and quality traits is not trivial due to heterogeneity of signalling mechanisms (physical, biochemical, and molecular), while species-specific variability adds an extra layer of complexity. Attempting to integrate and manipulate these disparate signals requires deeper understanding of how these signals are perceived and propagated, identification of points of convergence, and precise manipulation of critical nodes to obtain the desired outcomes. While we outline the current knowledge and potential traits that can be targeted by engineering these interactions, future research efforts will dictate our proximity to the desired traits.

## References

[eraf320-B1] Adamski NM, Anastasiou E, Eriksson S, O’Neill CM, Lenhard M. 2009. Local maternal control of seed size by KLUH/CYP78A5-dependent growth signaling. Proceedings of the National Academy of Sciences, USA 106, 20115–20120. 10.1073/pnas.0907024106 PMC2785301 19892740

[eraf320-B2] Ali MF, Shin J-M, Fatema U, Kurihara D, Berger F, Yuan L, Kawashima T. 2023. Cellular dynamics of endosperm development in *Arabidopsis thaliana*. Nature Plants 9, 330–342. 36646830 10.1038/s41477-022-01331-7

[eraf320-B3] Batista RA, Figueiredo DD, Santos-González J, Köhler C. 2019a. Auxin regulates endosperm cellularization in *Arabidopsis*. Genes & Development 33, 466–476. 30819818 10.1101/gad.316554.118 PMC6446538

[eraf320-B4] Batista RA, Moreno-Romero J, van Boven J, Qiu Y, Santos-González J, Figueiredo DD, Köhler C. 2019b. The MADS-box transcription factor PHERES1 controls imprinting in the endosperm by binding to domesticated transposons. eLife 8, 616698. 10.7554/eLife.50541 PMC6914339 31789592

[eraf320-B5] Batista RA, Köhler C. 2020. Genomic imprinting in plants-revisiting existing models. Genes & Development 34, 24–36. 31896690 10.1101/gad.332924.119 PMC6938664

[eraf320-B6] Bente H, Köhler C. 2024. Molecular basis and evolutionary drivers of endosperm-based hybridization barriers. Plant Physiology 195, 155–169. 38298124 10.1093/plphys/kiae050 PMC11060687

[eraf320-B7] Boccaccini A, Cimini S, Kazmi H, Lepri A, Longo C, Lorrai R, Vittorioso P. 2024. When size matters: new insights on how seed size can contribute to the early stages of plant development. Plants 13, 1793. 38999633 10.3390/plants13131793 PMC11244240

[eraf320-B8] Brown RC, Lemmon BE, Nguyen H, Olsen O-A. 1999. Development of endosperm in *Arabidopsis thaliana*. Sexual Plant Reproduction 12, 32–42.

[eraf320-B9] Cao J, Li G, Qu D, Li X, Wang Y. 2020. Into the seed: auxin controls seed development and grain yield. International Journal of Molecular Sciences 21, 1662. 32121296 10.3390/ijms21051662 PMC7084539

[eraf320-B10] Chen J, Lausser A, Dresselhaus T. 2014. Hormonal responses during early embryogenesis in maize. Biochemical Society Transactions 42, 325–331. 24646239 10.1042/BST20130260

[eraf320-B11] Cheng X, Pan M, E Z, Zhou Y, Niu B, Chen C. 2021. The maternally expressed polycomb group gene *OsEMF2a* is essential for endosperm cellularization and imprinting in rice. Plant Communications 2, 100092. 33511344 10.1016/j.xplc.2020.100092 PMC7816080

[eraf320-B12] Clark JK, Sheridan WF. 1991. Isolation and characterization of 51 embryo-specific mutations of maize. The Plant Cell 3, 935–951. 12324623 10.1105/tpc.3.9.935 PMC160061

[eraf320-B13] Costa LM, Marshall E, Tesfaye M, et al 2014. Central cell–derived peptides regulate early embryo patterning in flowering plants. Science 344, 168–172. 24723605 10.1126/science.1243005

[eraf320-B14] Creff A, Brocard L, Ingram G. 2015. A mechanically sensitive cell layer regulates the physical properties of the *Arabidopsis* seed coat. Nature Communications 6, 6382. 10.1038/ncomms7382 25702924

[eraf320-B15] Creff A, Brocard L, Joubès J, et al 2019. A stress-response-related inter-compartmental signalling pathway regulates embryonic cuticle integrity in *Arabidopsis*. PLoS Genetics 15, e1007847. 30998684 10.1371/journal.pgen.1007847 PMC6490923

[eraf320-B16] Creff A, Ali O, Bied C, Bayle V, Ingram G, Landrein B. 2023. Evidence that endosperm turgor pressure both promotes and restricts seed growth and size. Nature Communications 14, 67. 10.1038/s41467-022-35542-5 PMC9814827 36604410

[eraf320-B17] Debeaujon I, Nesi N, Perez P, Devic M, Grandjean O, Caboche M, Lepiniec L. 2003. Proanthocyanidin-accumulating cells in Arabidopsis testa: regulation of differentiation and role in seed development. The Plant Cell 15, 2514–2531. 14555692 10.1105/tpc.014043 PMC280558

[eraf320-B18] Doll NM, Depège-Fargeix N, Rogowsky PM, Widiez T. 2017. Signaling in early maize kernel development. Molecular Plant 10, 375–388. 28267956 10.1016/j.molp.2017.01.008

[eraf320-B19] Doll NM, Just J, Brunaud V, et al 2020a. Transcriptomics at maize embryo/endosperm interfaces identifies a transcriptionally distinct endosperm subdomain adjacent to the embryo scutellum. The Plant Cell 32, 833–852. 32086366 10.1105/tpc.19.00756 PMC7145466

[eraf320-B20] Doll NM, Royek S, Fujita S, et al 2020b. A two-way molecular dialogue between embryo and endosperm is required for seed development. Science 367, 431–435. 31974252 10.1126/science.aaz4131

[eraf320-B21] Dorcey E, Urbez C, Blázquez MA, Carbonell J, Perez-Amador MA. 2009. Fertilization-dependent auxin response in ovules triggers fruit development through the modulation of gibberellin metabolism in Arabidopsis. The Plant Journal 58, 318–332. 19207215 10.1111/j.1365-313X.2008.03781.x

[eraf320-B22] Doughty J, Aljabri M, Scott RJ. 2014. Flavonoids and the regulation of seed size in *Arabidopsis*. Biochemical Society Transactions 42, 364–369. 24646245 10.1042/BST20140040

[eraf320-B23] Dresselhaus T, Sprunck S, Wessel GM. 2016. Fertilization mechanisms in flowering plants. Current Biology 26, R125–R139. 26859271 10.1016/j.cub.2015.12.032 PMC4934421

[eraf320-B24] Figueiredo DD, Batista RA, Roszak PJ, Köhler C. 2015. Auxin production couples endosperm development to fertilization. Nature Plants 1, 15184. 27251719 10.1038/nplants.2015.184

[eraf320-B25] Figueiredo DD, Batista RA, Roszak PJ, Hennig L, Köhler C. 2016. Auxin production in the endosperm drives seed coat development in *Arabidopsis*. eLife 5, e20542. 27848912 10.7554/eLife.20542 PMC5135394

[eraf320-B26] Fiume E, Fletcher JC. 2012. Regulation of *Arabidopsis* embryo and endosperm development by the polypeptide signaling molecule CLE8. The Plant Cell 24, 1000–1012. 22427333 10.1105/tpc.111.094839 PMC3336133

[eraf320-B27] Fiume E, Guyon V, Remoué C, Magnani E, Miquel M, Grain D, Lepiniec L. 2016. TWS1, a novel small protein, regulates various aspects of seed and plant development. Plant Physiology 172, 1732–1745. 27613850 10.1104/pp.16.00915 PMC5100777

[eraf320-B28] Florez-Rueda AM, Miguel CM, Figueiredo DD. 2024a. Comparative transcriptomics of seed nourishing tissues: uncovering conserved and divergent pathways in seed plants. The Plant Journal 119, 1134–1157. 38709819 10.1111/tpj.16786

[eraf320-B29] Florez-Rueda AM, Scharmann M, De Souza LP, Fernie AR, Bachelier JB, Figueiredo DD. 2024b. Genomic imprinting in an early-diverging angiosperm reveals ancient mechanisms for seed initiation in flowering plants. bioRxiv, doi: 10.1101/2024.09.18.613646. [Preprint]. 10.1111/nph.70776 PMC12825398 41315881

[eraf320-B30] Forestan C, Meda S, Varotto S. 2010. ZmPIN1-mediated auxin transport is related to cellular differentiation during maize embryogenesis and endosperm development. Plant Physiology 152, 1373–1390. 20044449 10.1104/pp.109.150193 PMC2832270

[eraf320-B31] Fouquet R, Martin F, Fajardo DS, Gault CM, Gómez E, Tseung C-W, Policht T, Hueros G, Settles AM. 2011. Maize *Rough Endosperm3* encodes an RNA splicing factor required for endosperm cell differentiation and has a nonautonomous effect on embryo development. The Plant Cell 23, 4280–4297. 22138152 10.1105/tpc.111.092163 PMC3269866

[eraf320-B32] Fukuda H, Higashiyama T. 2011. Diverse functions of plant peptides: entering a new phase. Plant & Cell Physiology 52, 1–4. 21248365 10.1093/pcp/pcq193

[eraf320-B33] Garcia D, Saingery V, Chambrier P, Mayer U, Jürgens G, Berger F. 2003. Arabidopsis *haiku* mutants reveal new controls of seed size by endosperm. Plant Physiology 131, 1661–1670. 12692325 10.1104/pp.102.018762 PMC166922

[eraf320-B34] Garcia D, Fitz Gerald JN, Berger F. 2005. Maternal control of integument cell elongation and zygotic control of endosperm growth are coordinated to determine seed size in Arabidopsis. The Plant Cell 17, 52–60. 15598800 10.1105/tpc.104.027136 PMC544489

[eraf320-B35] Gehring M, Missirian V, Henikoff S. 2011. Genomic analysis of parent-of-origin allelic expression in *Arabidopsis thaliana* seeds. PLoS One 6, e23687. 21858209 10.1371/journal.pone.0023687 PMC3157454

[eraf320-B36] Gooh K, Ueda M, Aruga K, Park J, Arata H, Higashiyama T, Kurihara D. 2015. Live-cell imaging and optical manipulation of *Arabidopsis* early embryogenesis. Developmental Cell 34, 242–251. 26166301 10.1016/j.devcel.2015.06.008

[eraf320-B37] Gusti A, Baumberger N, Nowack M, Pusch S, Eisler H, Potuschak T, De Veylder L, Schnittger A, Genschik P. 2009. The *Arabidopsis thaliana* F-box protein FBL17 is essential for progression through the second mitosis during pollen development. PLoS One 4, e4780. 19277118 10.1371/journal.pone.0004780 PMC2651519

[eraf320-B38] Haughn G, Chaudhury A. 2005. Genetic analysis of seed coat development in *Arabidopsis*. Trends in Plant Science 10, 472–477. 16153880 10.1016/j.tplants.2005.08.005

[eraf320-B39] Hehenberger E, Kradolfer D, Köhler C. 2012. Endosperm cellularization defines an important developmental transition for embryo development. Development 139, 2031–2039. 22535409 10.1242/dev.077057

[eraf320-B40] Hong SK, Kitano H, Satoh H, Nagato Y. 1996. How is embryo size genetically regulated in rice? Development 122, 2051–2058. 8681786 10.1242/dev.122.7.2051

[eraf320-B41] Hsieh TF, Shin J, Uzawa R, et al 2011. Regulation of imprinted gene expression in *Arabidopsis* endosperm. Proceedings of the National Academy of Sciences, USA 108, 1755–1762. 10.1073/pnas.1019273108 PMC3033266 21257907

[eraf320-B42] Huc J, Dziasek K, Pachamuthu K, Woh T, Köhler C, Borges F. 2022. Bypassing reproductive barriers in hybrid seeds using chemically induced epimutagenesis. The Plant Cell 34, 989–1001. 34792584 10.1093/plcell/koab284 PMC8894923

[eraf320-B43] Ingouff M, Jullien PE, Berger F. 2006. The female gametophyte and the endosperm control cell proliferation and differentiation of the seed coat in Arabidopsis. The Plant Cell 18, 3491–3501. 17172356 10.1105/tpc.106.047266 PMC1785409

[eraf320-B44] Ingram G, Gutierrez-Marcos J. 2015. Peptide signalling during angiosperm seed development. Journal of Experimental Botany 66, 5151–5159. 26195729 10.1093/jxb/erv336

[eraf320-B45] Ingram G, Nawrath C. 2017. The roles of the cuticle in plant development: organ adhesions and beyond. Journal of Experimental Botany 68, 5307–5321. 28992283 10.1093/jxb/erx313

[eraf320-B46] Ito T, Okada K, Fukazawa J, Takahashi Y. 2018. DELLA-dependent and -independent gibberellin signaling. Plant Signaling & Behavior 13, e1445933. 29485381 10.1080/15592324.2018.1445933 PMC5927702

[eraf320-B47] Iwakawa H, Shinmyo A, Sekine M. 2006. Arabidopsis *CDKA;1*, a *cdc2* homologue, controls proliferation of generative cells in male gametogenesis. The Plant Journal 45, 819–831. 16460514 10.1111/j.1365-313X.2005.02643.x

[eraf320-B48] Jeng TL, Shih YJ, Ho PT, Lai CC, Lin YW, Wang CS, Sung JM. 2012. γ-Oryzanol, tocol and mineral compositions in different grain fractions of giant embryo rice mutants: nutrient composition in giant embryo rice mutants. Journal of the Science of Food and Agriculture 92, 1468–1474. 22131276 10.1002/jsfa.4728

[eraf320-B49] Jiang W-B, Huang H-Y, Hu Y-W, Zhu S-W, Wang Z-Y, Lin W-H. 2013. Brassinosteroid regulates seed size and shape in Arabidopsis. Plant Physiology 162, 1965–1977. 23771896 10.1104/pp.113.217703 PMC3729775

[eraf320-B50] Kasahara RD, Notaguchi M, Nagahara S, Suzuki T, Susaki D, Honma Y, Maruyama D, Higashiyama T. 2016. Pollen tube contents initiate ovule enlargement and enhance seed coat development without fertilization. Science Advances 2, e1600554. 27819041 10.1126/sciadv.1600554 PMC5091356

[eraf320-B51] Kim JY, Seo WD, Park D-S, et al 2013. Comparative studies on major nutritional components of black waxy rice with giant embryos and its rice bran. Food Science and Biotechnology 22, 121–128.

[eraf320-B52] Koh H-J, Heu M-H, McCouch SR. 1996. Molecular mapping of the *ge*^s^ gene controlling the super-giant embryo character in rice (*Oryza sativa* L.). Theoretical and Applied Genetics 93–93, 257–261. 10.1007/BF00225754 24162226

[eraf320-B53] Kradolfer D, Wolff P, Jiang H, Siretskiy A, Köhler C. 2013. An imprinted gene underlies postzygotic reproductive isolation in *Arabidopsis thaliana*. Developmental Cell 26, 525–535. 24012484 10.1016/j.devcel.2013.08.006

[eraf320-B54] Krzyszton M, Sacharowski SP, Manjunath VH, Muter K, Bokota G, Wang C, Plewczyński D, Dobisova T, Swiezewski S. 2024. Dormancy heterogeneity among *Arabidopsis thaliana* seeds is linked to individual seed size. Plant Communications 5, 100732. 37828740 10.1016/j.xplc.2023.100732 PMC10873894

[eraf320-B55] Lafon-Placette C, Köhler C. 2014. Embryo and endosperm, partners in seed development. Current Opinion in Plant Biology 17, 64–69. 24507496 10.1016/j.pbi.2013.11.008

[eraf320-B56] Lee G, Piao R, Lee Y, et al 2019. Identification and characterization of *LARGE EMBRYO*, a new gene controlling embryo size in rice (*Oryza sativa* L.). Rice 12, 22. 30972509 10.1186/s12284-019-0277-y PMC6458227

[eraf320-B57] Li C, Liu J, Zhang L, Li T, Li H, Liu B, Zhao T. 2024. OsDA1 positively regulates grain width in rice. The Crop Journal 12, 92–101.

[eraf320-B58] Lima RB, Figueiredo DD. 2024. Sex on steroids: how brassinosteroids shape reproductive development in plants. Plant & Cell Physiology 65, 1581–1600. 38668644 10.1093/pcp/pcae050 PMC11558549

[eraf320-B59] Lima RB, Pankaj R, Ehlert ST, Finger P, Fröhlich A, Bayle V, Landrein B, Sampathkumar A, Figueiredo DD. 2024. Seed coat-derived brassinosteroid signaling regulates endosperm development. Nature Communications 15, 9352. 10.1038/s41467-024-53671-x PMC11522626 39472566

[eraf320-B60] Lin Z, Zhang X, Yang X, Li G, Tang S, Wang S, Ding Y, Liu Z. 2014. Proteomic analysis of proteins related to rice grain chalkiness using iTRAQ and a novel comparison system based on a notched-belly mutant with white-belly. BMC Plant Biology 14, 163. 24924297 10.1186/1471-2229-14-163 PMC4072481

[eraf320-B61] Lin Z, Zheng D, Zhang X, Wang Z, Lei J, Liu Z, Li G, Wang S, Ding Y. 2016. Chalky part differs in chemical composition from translucent part of japonica rice grains as revealed by a notched-belly mutant with white-belly. Journal of the Science of Food and Agriculture 96, 3937–3943. 27166835 10.1002/jsfa.7793 PMC5089642

[eraf320-B62] Lin Z, Wang Z, Zhang X, Liu Z, Li G, Wang S, Ding Y. 2017. Complementary proteome and transcriptome profiling in developing grains of a notched-belly rice mutant reveals key pathways involved in chalkiness formation. Plant & Cell Physiology 58, 560–573. 28158863 10.1093/pcp/pcx001 PMC5444571

[eraf320-B63] Liu H, Luo Q, Tan C, Song J, Zhang T, Men S. 2023. Biosynthesis- and transport-mediated dynamic auxin distribution during seed development controls seed size in Arabidopsis. The Plant Journal 113, 1259–1277. 36648165 10.1111/tpj.16109

[eraf320-B64] Liu X, Nakajima KP, Adhikari PB, et al 2025. Fertilization-dependent phloem end gate regulates seed size. Current Biology 35, 2049–2063.e3. 40199323 10.1016/j.cub.2025.03.033

[eraf320-B65] Luo M, Dennis ES, Berger F, Peacock WJ, Chaudhury A. 2005. *MINISEED3* (*MINI3*), a WRKY family gene, and *HAIKU2* (*IKU2*), a leucine-rich repeat (*LRR*) *KINASE* gene, are regulators of seed size in *Arabidopsis*. Proceedings of the National Academy of Sciences, USA 102, 17531–17536. 10.1073/pnas.0508418102 PMC1297679 16293693

[eraf320-B66] Martinez G, Wolff P, Wang Z, Moreno-Romero J, Santos-González J, Conze LL, DeFraia C, Slotkin RK, Köhler C. 2018. Paternal easiRNAs regulate parental genome dosage in *Arabidopsis*. Nature Genetics 50, 193–198. 29335548 10.1038/s41588-017-0033-4

[eraf320-B67] Mimura M, Kudo T, Wu S, McCarty DR, Suzuki M. 2018. Autonomous and non-autonomous functions of the maize *Shohai1* gene, encoding a RWP - RK putative transcription factor, in regulation of embryo and endosperm development. The Plant Journal 95, 892–908. 10.1111/tpj.13996 29901832

[eraf320-B68] Muthusamy M, Pandian S, Shin E-K, An H-K, Sohn S-I. 2024. Unveiling the imprinted dance: how parental genomes orchestrate seed development and hybrid success. Frontiers in Plant Science 15, 1455685. 39399543 10.3389/fpls.2024.1455685 PMC11466797

[eraf320-B69] Nagasawa N, Hibara K, Heppard EP, Vander Velden KA, Luck S, Beatty M, Nagato Y, Sakai H. 2013. *GIANT EMBRYO* encodes CYP78A13, required for proper size balance between embryo and endosperm in rice. The Plant Journal 75, 592–605. 23621326 10.1111/tpj.12223

[eraf320-B70] Niu B, Zhang Z, Zhang J, Zhou Y, Chen C. 2021. The rice LEC1-like transcription factor OsNF-YB9 interacts with SPK, an endosperm-specific sucrose synthase protein kinase, and functions in seed development. The Plant Journal 106, 1233–1246. 33721364 10.1111/tpj.15230

[eraf320-B71] Ohto M, Fischer RL, Goldberg RB, Nakamura K, Harada JJ. 2005. Control of seed mass by *APETALA2*. Proceedings of the National Academy of Sciences, USA 102, 3123–3128. 10.1073/pnas.0409858102 PMC549491 15708976

[eraf320-B72] Ohto MA, Floyd SK, Fischer RL, Goldberg RB, Harada JJ. 2009. Effects of APETALA2 on embryo, endosperm, and seed coat development determine seed size in Arabidopsis. Sexual Plant Reproduction 22, 277–289. 20033449 10.1007/s00497-009-0116-1 PMC2796121

[eraf320-B73] Olsen O-A . 2001. Endosperm development: cellularization and cell fate specification. Annual Review of Plant Physiology and Plant Molecular Biology 52, 233–267. 10.1146/annurev.arplant.52.1.233 11337398

[eraf320-B74] Olsen O-A . 2004. Nuclear endosperm development in cereals and *Arabidopsis thaliana*. The Plant Cell 16, S214–S227. 15010513 10.1105/tpc.017111 PMC2643391

[eraf320-B75] Pankaj R, Lima RB, Luo G-Y, Ehlert S, Del Toro-de León G, Bente H, Finger P, Sato H, Figueiredo DD. 2023. BRI1-mediated removal of seed coat H3K27me3 marks is a brassinosteroid-independent process. bioRxiv, doi: 10.1101/2023.12.07.569203. [Preprint]. 10.1093/plcell/koag182 PMC13389951 42287738

[eraf320-B76] Pankaj R, Lima RB, Figueiredo DD. 2025. Hormonal regulation and crosstalk during early endosperm and seed coat development. Plant Reproduction 38, 5. 10.1007/s00497-024-00516-8 PMC11671439 39724433

[eraf320-B77] Peer WA, Bandyopadhyay A, Blakeslee JJ, Makam SN, Chen RJ, Masson PH, Murphy AS. 2004. Variation in expression and protein localization of the PIN family of auxin efflux facilitator proteins in flavonoid mutants with altered auxin transport in *Arabidopsis thaliana*. The Plant Cell 16, 1898–1911. 15208397 10.1105/tpc.021501 PMC514169

[eraf320-B78] Peer WA, Murphy AS. 2007. Flavonoids and auxin transport: modulators or regulators? Trends in Plant Science 12, 556–563. 18198522 10.1016/j.tplants.2007.10.003

[eraf320-B79] Peng X, Sun M-X. 2018. The suspensor as a model system to study the mechanism of cell fate specification during early embryogenesis. Plant Reproduction 31, 59–65. 29473100 10.1007/s00497-018-0326-5 PMC5845063

[eraf320-B80] Pennington PD, Costa LM, Gutierrez-Marcos JF, Greenland AJ, Dickinson HG. 2008. When genomes collide: aberrant seed development following maize interploidy crosses. Annals of Botany 101, 833–843. 18276791 10.1093/aob/mcn017 PMC2710208

[eraf320-B81] Picard CL, Povilus RA, Williams BP, Gehring M. 2021. Transcriptional and imprinting complexity in *Arabidopsis* seeds at single-nucleus resolution. Nature Plants 7, 730–738. 34059805 10.1038/s41477-021-00922-0 PMC8217372

[eraf320-B82] Pires ND . 2014. Seed evolution: parental conflicts in a multi-generational household. BioMolecular Concepts 5, 71–86. 25372743 10.1515/bmc-2013-0034

[eraf320-B83] Reiser L, Fischer RL. 1993. The ovule and the embryo sac. The Plant Cell 5, 1291–1301. 12271029 10.1105/tpc.5.10.1291 PMC160362

[eraf320-B84] Robert HS, Grones P, Stepanova AN, Robles LM, Lokerse AS, Alonso JM, Weijers D, Friml J. 2013. Local auxin sources orient the apical-basal axis in *Arabidopsis* embryos. Current Biology 23, 2506–2512. 24291089 10.1016/j.cub.2013.09.039

[eraf320-B85] Robert HS, Park C, Gutièrrez CL, et al 2018. Maternal auxin supply contributes to early embryo patterning in *Arabidopsis*. Nature Plants 4, 548–553. 30013211 10.1038/s41477-018-0204-z PMC6076996

[eraf320-B86] Ron M, Saez MA, Williams LE, Fletcher JC, McCormick S. 2010. Proper regulation of a sperm-specific cis-nat-siRNA is essential for double fertilization in *Arabidopsis*. Genes & Development 24, 1010–1021. 20478994 10.1101/gad.1882810 PMC2867206

[eraf320-B87] Roszak P, Köhler C. 2011. Polycomb group proteins are required to couple seed coat initiation to fertilization. Proceedings of the National Academy of Sciences, USA 108, 20826–20831. 10.1073/pnas.1117111108 PMC3251106 22143805

[eraf320-B88] Royek S, Bayer M, Pfannstiel J, Pleiss J, Ingram G, Stintzi A, Schaller A. 2022. Processing of a plant peptide hormone precursor facilitated by posttranslational tyrosine sulfation. Proceedings of the National Academy of Sciences, USA 119, e2201195119. 10.1073/pnas.2201195119 PMC9169856 35412898

[eraf320-B89] Sakata M, Seno M, Matsusaka H, et al 2016. Development and evaluation of rice giant embryo mutants for high oil content originated from a high-yielding cultivar ‘Mizuhochikara’. Breeding Science 66, 425–433. 27436953 10.1270/jsbbs.15135 PMC4902460

[eraf320-B90] San-Bento R, Farcot E, Galletti R, Creff A, Ingram G. 2014. Epidermal identity is maintained by cell–cell communication via a universally active feedback loop in *Arabidopsis thaliana*. The Plant Journal 77, 46–58. 24147836 10.1111/tpj.12360

[eraf320-B91] Satyaki PRV, Gehring M. 2019. Paternally acting canonical RNA-directed DNA methylation pathway genes sensitize *Arabidopsis* endosperm to paternal genome dosage. The Plant Cell 31, 1563–1578. 31064867 10.1105/tpc.19.00047 PMC6635864

[eraf320-B92] Schmid MW, Schmidt A, Grossniklaus U. 2015. The female gametophyte: an emerging model for cell type-specific systems biology in plant development. Frontiers in Plant Science 6, 907. 26579157 10.3389/fpls.2015.00907 PMC4630298

[eraf320-B93] Schneitz K, Hülskamp M, Pruitt RE. 1995. Wild-type ovule development in *Arabidopsis thaliana*: a light microscope study of cleared whole-mount tissue. The Plant Journal 7, 731–749.

[eraf320-B94] Scott RJ, Spielman M, Bailey J, Dickinson HG. 1998. Parent-of-origin effects on seed development in *Arabidopsis thaliana*. Development 125, 3329–3341. 9693137 10.1242/dev.125.17.3329

[eraf320-B95] Sekine D, Ohnishi T, Furuumi H, Ono A, Yamada T, Kurata N, Kinoshita T. 2013. Dissection of two major components of the post-zygotic hybridization barrier in rice endosperm. The Plant Journal 76, 792–799. 24286595 10.1111/tpj.12333

[eraf320-B96] Senoura T, Sakashita E, Kobayashi T, Takahashi M, Aung MS, Masuda H, Nakanishi H, Nishizawa NK. 2017. The iron-chelate transporter OsYSL9 plays a role in iron distribution in developing rice grains. Plant Molecular Biology 95, 375–387. 28871478 10.1007/s11103-017-0656-y

[eraf320-B97] Shi C, Luo P, Du Y-T, et al 2019. Maternal control of suspensor programmed cell death via gibberellin signaling. Nature Communications 10, 3484. 10.1038/s41467-019-11476-3 PMC6677759 31375676

[eraf320-B98] Song J, Xie X, Chen C, et al 2021. LEAFY COTYLEDON1 expression in the endosperm enables embryo maturation in Arabidopsis. Nature Communications 12, 3963. 10.1038/s41467-021-24234-1 PMC8233312 34172749

[eraf320-B99] Stoute AI, Varenko V, King GJ, Scott RJ, Kurup S. 2012. Parental genome imbalance in *Brassica oleracea* causes asymmetric triploid block. The Plant Journal 71, 503–516. 22679928 10.1111/j.1365-313X.2012.05015.x

[eraf320-B100] Tanaka H, Onouchi H, Kondo M, Hara-Nishimura I, Nishimura M, Machida C, Machida Y. 2001. A subtilisin-like serine protease is required for epidermal surface formation in *Arabidopsis* embryos and juvenile plants. Development 128, 4681–4689. 11731449 10.1242/dev.128.23.4681

[eraf320-B101] Tao Y, An L, Xiao F, Li G, Ding Y, Paul MJ, Liu Z. 2022a. Integration of embryo–endosperm interaction into a holistic and dynamic picture of seed development using a rice mutant with notched-belly kernels. The Crop Journal 10, 729–742.

[eraf320-B102] Tao Y, Mohi Ud Din A, An L, Chen H, Li G, Ding Y, Liu Z. 2022b. Metabolic disturbance induced by the embryo contributes to the formation of chalky endosperm of a notched-belly rice mutant. Frontiers in Plant Science 12, 760597. 35069619 10.3389/fpls.2021.760597 PMC8767064

[eraf320-B103] Tedeschi F, Rizzo P, Rutten T, Altschmied L, Bäumlein H. 2017. RWP-RK domain-containing transcription factors control cell differentiation during female gametophyte development in Arabidopsis. New Phytologist 213, 1909–1924. 27870062 10.1111/nph.14293

[eraf320-B104] Tonosaki K, Ono A, Kunisada M, et al 2021. Mutation of the imprinted gene *OsEMF2a* induces autonomous endosperm development and delayed cellularization in rice. The Plant cell 33, 85–103. 33751094 10.1093/plcell/koaa006 PMC8136911

[eraf320-B105] Tsuwamoto R, Fukuoka H, Takahata Y. 2008. *GASSHO1* and *GASSHO2* encoding a putative leucine-rich repeat transmembrane-type receptor kinase are essential for the normal development of the epidermal surface in Arabidopsis embryos. The Plant Journal 54, 30–42. 18088309 10.1111/j.1365-313X.2007.03395.x

[eraf320-B106] Van Ekelenburg YS, Hornslien KS, Van Hautegem T, Fendrych M, Van Isterdael G, Bjerkan KN, Miller JR, Nowack MK, Grini PE. 2023. Spatial and temporal regulation of parent-of-origin allelic expression in the endosperm. Plant Physiology 191, 986–1001. 36437711 10.1093/plphys/kiac520 PMC9922421

[eraf320-B107] Vijayan A, Tofanelli R, Strauss S, Cerrone L, Wolny A, Strohmeier J, Kreshuk A, Hamprecht FA, Smith RS, Schneitz K. 2021. A digital 3D reference atlas reveals cellular growth patterns shaping the *Arabidopsis* ovule. eLife 10, e63262. 33404501 10.7554/eLife.63262 PMC7787667

[eraf320-B108] Wang A, Garcia D, Zhang H, Feng K, Chaudhury A, Berger F, Peacock WJ, Dennis ES, Luo M. 2010. The VQ motif protein IKU1 regulates endosperm growth and seed size in Arabidopsis. The Plant Journal 63, 670–679. 20545893 10.1111/j.1365-313X.2010.04271.x

[eraf320-B109] Wang J, Tang M, Chen S, et al 2017. Down-regulation of BnDA1, whose gene locus is associated with the seeds weight, improves the seeds weight and organ size in *Brassica napus*. Plant Biotechnology Journal 15, 1024–1033. 28097785 10.1111/pbi.12696 PMC5506660

[eraf320-B110] Wang X, Sun J, Yi Z, Dong S. 2025. Effects of seed size on soybean performance: germination, growth, stress resistance, photosynthesis, and yield. BMC Plant Biology 25, 219. 39966748 10.1186/s12870-025-06224-3 PMC11834689

[eraf320-B111] Weber H, Borisjuk L, Wobus U. 1997. Sugar import and metabolism during seed development. Trends in Plant Science 2, 169–174.

[eraf320-B112] Weijers D, van Hamburg JP, Rijn EV, Hooykaas PJ, Offringa R. 2003. Diphtheria toxin-mediated cell ablation reveals interregional communication during Arabidopsis seed development. Plant Physiology 133, 1882–1892. 14605218 10.1104/pp.103.030692 PMC300741

[eraf320-B113] Wendrich JR, Weijers D. 2013. The Arabidopsis embryo as a miniature morphogenesis model. New Phytologist 199, 14–25. 23590679 10.1111/nph.12267

[eraf320-B114] Wolff P, Weinhofer I, Seguin J, Roszak P, Beisel C, Donoghue MT, Spillane C, Nordborg M, Rehmsmeier M, Köhler C. 2011. High-resolution analysis of parent-of-origin allelic expression in the Arabidopsis endosperm. PLoS Genetics 7, e1002126. 21698132 10.1371/journal.pgen.1002126 PMC3116908

[eraf320-B115] Wolff P, Jiang H, Wang G, Santos-González J, Köhler C. 2015. Paternally expressed imprinted genes establish postzygotic hybridization barriers in *Arabidopsis thaliana*. eLife 4, e10074. 26344545 10.7554/eLife.10074 PMC4589659

[eraf320-B116] Wu C-C, Diggle PK, Friedman WE. 2013. Kin recognition within a seed and the effect of genetic relatedness of an endosperm to its compatriot embryo on maize seed development. Proceedings of the National Academy of Sciences, USA 110, 2217–2222. 10.1073/pnas.1220885110 PMC3568322 23345441

[eraf320-B117] Wu X, Cai X, Zhang B, Wu S, Wang R, Li N, Li Y, Sun Y, Tang W. 2022. ERECTA regulates seed size independently of its intracellular domain via MAPK-DA1-UBP15 signaling. The Plant Cell 34, 3773–3789. 35848951 10.1093/plcell/koac194 PMC9516062

[eraf320-B118] Xie G, Li Z, Ran Q, Wang H, Zhang J. 2018. Over-expression of mutated *ZmDA1* or *ZmDAR1* gene improves maize kernel yield by enhancing starch synthesis. Plant Biotechnology Journal 16, 234–244. 28557341 10.1111/pbi.12763 PMC5785342

[eraf320-B119] Xing Q, Creff A, Waters A, Tanaka H, Goodrich J, Ingram GC. 2013. ZHOUPI controls embryonic cuticle formation via a signalling pathway involving the subtilisin protease ABNORMAL LEAF-SHAPE1 and the receptor kinases GASSHO1 and GASSHO2. Development 140, 770–779. 23318634 10.1242/dev.088898

[eraf320-B120] Xiong H, Wang W, Sun M-X. 2021. Endosperm development is an autonomously programmed process independent of embryogenesis. The Plant Cell 33, 1151–1160. 33793916 10.1093/plcell/koab007

[eraf320-B121] Xu F, Fang J, Ou S, et al 2015. Variations in *CYP78A13* coding region influence grain size and yield in rice. Plant, Cell & Environment 38, 800–811. 10.1111/pce.12452 25255828

[eraf320-B122] Xu W, Sato H, Bente H, Santos-González J, Köhler C. 2023. Endosperm cellularization failure induces a dehydration-stress response leading to embryo arrest. The Plant Cell 35, 874–888. 36427255 10.1093/plcell/koac337 PMC9940880

[eraf320-B123] Yang W, Gao M, Yin X, et al 2013. Control of rice embryo development, shoot apical meristem maintenance, and grain yield by a novel cytochrome P450. Molecular Plant 6, 1945–1960. 23775595 10.1093/mp/sst107

[eraf320-B124] Yang Y, Chu C, Qian Q, Tong H. 2024. Leveraging brassinosteroids towards the next green revolution. Trends in Plant Science 29, 86–98. 37805340 10.1016/j.tplants.2023.09.005

[eraf320-B125] Zhang H, Luo M, Johnson SD, Zhu X, Liu L, Huang F, Liu Y, Xu P, Wu X. 2016. Parental genome imbalance causes post-zygotic seed lethality and deregulates imprinting in rice. Rice 9, 43. 27568375 10.1186/s12284-016-0115-4 PMC5002275

[eraf320-B126] Zhang L, Hu P, Tang S, Zhao H, Wu D. 2005. Comparative studies on major nutritional components of rice with a giant embryo and a normal embryo. Journal of Food Biochemistry 29, 653–661.

[eraf320-B127] Zhang Y, Zhang B, Yan D, et al 2011. Two Arabidopsis cytochrome P450 monooxygenases, CYP714A1 and CYP714A2, function redundantly in plant development through gibberellin deactivation. The Plant Journal 67, 342–353. 21457373 10.1111/j.1365-313X.2011.04596.x

[eraf320-B128] Zhang Y, Maruyama D, Toda E, Kinoshita A, Okamoto T, Mitsuda N, Takasaki H, Ohme-Takagi M. 2023. Transcriptome analyses uncover reliance of endosperm gene expression on *Arabidopsis* embryonic development. FEBS Letters 597, 407–417. 36645411 10.1002/1873-3468.14570

[eraf320-B129] Zhao P, Zhou X, Zhang L, Wang W, Ma L, Yang L, Peng X, Bozhkov PV, Sun M. 2013. A bipartite molecular module controls cell death activation in the basal cell lineage of plant embryos. PLoS Biology 11, e1001655. 24058297 10.1371/journal.pbio.1001655 PMC3769231

[eraf320-B130] Zheng X, Li Q, Li C, An D, Xiao Q, Wang W, Wu Y. 2019. Intra-kernel reallocation of proteins in maize Depends on VP1-mediated scutellum development and nutrient assimilation. The Plant Cell 31, 2613–2635. 31530735 10.1105/tpc.19.00444 PMC6881121

[eraf320-B131] Zhiguo E, Li T, Zhang H, Liu Z, Deng H, Sharma S, Wei X, Wang L, Niu B, Chen C. 2018. A group of nuclear factor Y transcription factors are sub-functionalized during endosperm development in monocots. Journal of Experimental Botany 69, 2495–2510. 29514259 10.1093/jxb/ery087 PMC5920288

[eraf320-B132] Zhou C, Lin Q, Ren Y, et al 2023. A CYP78As–small grain4–coat protein complex II pathway promotes grain size in rice. The Plant Cell 35, 4325–4346. 37738653 10.1093/plcell/koad239 PMC10689148

[eraf320-B133] Zhou Y, Zhang X, Kang X, Zhao X, Zhang X, Ni M. 2009. SHORT HYPOCOTYL UNDER BLUE1 associates with *MINISEED3* and *HAIKU2* promoters in vivo to regulate *Arabidopsis* seed development. The Plant Cell 21, 106–117. 19141706 10.1105/tpc.108.064972 PMC2648090

[eraf320-B134] Zumajo-Cardona C, Aguirre M, Castillo-Bravo R, Mizzotti C, Di Marzo M, Banfi C, Mendes MA, Spillane C, Colombo L, Ezquer I. 2023. Maternal control of triploid seed development by the TRANSPARENT TESTA 8 (TT8) transcription factor in *Arabidopsis thaliana*. Scientific Reports 13, 1316. 36693864 10.1038/s41598-023-28252-5 PMC9873634

